# SSUplex: fast, both-strand extraction and origin-sorting of small-subunit rRNA for environmental DNA metabarcoding

**DOI:** 10.1093/bioadv/vbag318

**Published:** 2026-10-23

**Authors:** Aaron O’Brien, Juan Vargas Botia, Isabel Acuña, Franko Restovic, Patricio Martínez, Pilar Parada

**Affiliations:** Centro de Biotecnología de Sistemas, Universidad Andrés Bello, Santiago, 7780274, Chile; Centro de Biotecnología de Sistemas, Universidad Andrés Bello, Santiago, 7780274, Chile; Centro de Biotecnología de Sistemas, Universidad Andrés Bello, Santiago, 7780274, Chile; Centro de Biotecnología de Sistemas, Universidad Andrés Bello, Santiago, 7780274, Chile; Centro de Biotecnología de Sistemas, Universidad Andrés Bello, Santiago, 7780274, Chile; Centro de Biotecnología de Sistemas, Universidad Andrés Bello, Santiago, 7780274, Chile

## Abstract

**Motivation:**

Long-read sequencing has made full-length small-subunit ribosomal RNA metabarcoding routine, but broadly conserved primers also amplify organellar and cross-domain sequences, which distort profiles when sent to a target-domain classifier.

**Results:**

SSUplex detects small-subunit ribosomal RNA on both strands with conserved-region profile hidden Markov models, assigns each read to one of five origins (bacteria, archaea, eukaryota, mitochondria, chloroplast) and extracts the gene region. It reimplements Metaxa2’s extraction step but ranks origins by summed rather than mean per-region score. On 5000 ZymoBIOMICS Nanopore reads, this raised accuracy from 54.9% for Metaxa2’s extraction step to 95.9%, against 99.96% for Metaxa2’s full pipeline with similarity-search classification. On reads simulated from sources outside the Metaxa2 database, SSUplex was 93 to 98% accurate for bacteria-dominated and plant-root-like compositions (Metaxa2’s full pipeline: 96 to 99%), with mitochondria the weakest origin. In a rice-root 16S sample, about 40% of reads were organellar. SSUplex ran 3.2× faster than Metaxa2’s extraction step from 200 000 reads upward, with about 45% less peak memory, and 65 to 134× faster than Metaxa2’s full pipeline.

**Availability and implementation:**

SSUplex is implemented in Rust and freely available at https://github.com/ayobi/ssuplex and from Bioconda; version 0.2.0 is archived at https://doi.org/10.5281/zenodo.23246210.

## 1 Introduction

Broadly conserved rRNA primers make small-subunit (SSU; 16S/18S) rRNA metabarcoding powerful but also co-amplify mitochondrial, chloroplast and cross-domain SSU. In host- and plant-associated samples these reads can be abundant and are misclassified if sent directly to a target-domain reference database, biasing relative abundance, richness and diversity estimates. Sorting reads by biological origin before classification is therefore an important preprocessing step.

*Metaxa2* (Bengtsson-Palme *et al*. 2015) detects SSU and large-subunit (LSU) rRNA genes with profile hidden Markov models (HMMs), assigns an origin from the HMM scores and can then classify the extracted sequences with BLAST; its final origin calls draw on both steps. SSUplex reimplements the HMM-based detection, origin-assignment and extraction step in Rust, scans both strands and leaves taxonomy to classifiers built for each marker (Fig. 1). RNAmmer (Lagesen *et al.* 2007), barrnap (Seemann 2018) and SortMeRNA (Kopylova *et al.* 2012) address adjacent tasks, rRNA annotation or rRNA filtering, rather than per-read origin assignment across domains and organelles.

**Figure 1:**
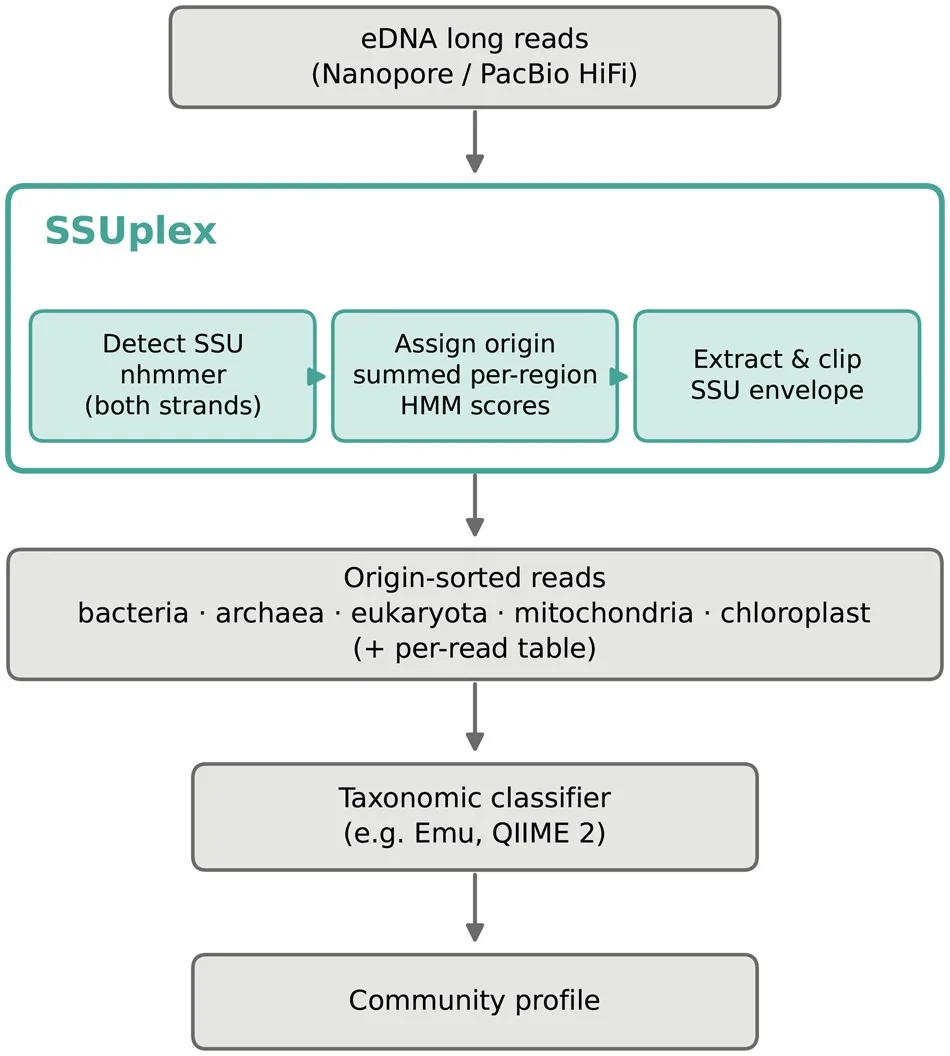
SSUplex workflow. Reads are scanned on both strands with nhmmer, assigned to one of five origins from summed per-region HMM scores, clipped to the SSU envelope and written as origin-sorted reads for marker-specific taxonomic classifiers such as Emu (Curry *et al.* 2022) or QIIME 2 (Bolyen *et al.* 2019).

## 2 Implementation

SSUplex reads FASTA input, optionally gzip-compressed, and searches it with nhmmer from HMMER 3 (Eddy 2011) against origin-specific profiles of conserved SSU regions from the Metaxa2 SSU database, with Metaxa2’s two mitochondrial sets combined. nhmmer scans both strands, so reads need no orientation step; hits with an E-value above 10^−5^ are discarded.

For each read and origin, SSUplex keeps at most one hit per conserved-region profile and summarizes the hits as a region count, a summed bit score and a mean bit score. By default the origin with the highest summed score is chosen; —rank mean and—rank count select the other statistics, which mirror Metaxa2’s selection priorities (the mean is Metaxa2’s default). A read is reported as unclassified when the chosen origin matches fewer than—min-domains regions (default 1) or its mean score is below—min-score. Accepted reads are clipped to the envelope of their hits and written to one FASTA file per origin, with a per-read table.

SSUplex is a single compiled binary whose only runtime dependency is nhmmer, and it can be installed from Bioconda. Continuous integration runs unit, end-to-end and installation tests, including the documented quick start, on Linux and macOS.

## 3 Results and discussion

We compared SSUplex with Metaxa2 v2.2.3, both using HMMER 3.4. Metaxa2 was scored at two stages: the origin from its HMM step, which SSUplex reimplements, and its final origin after BLAST classification. Accuracy counts reads that a method leaves undetected, unclassified or uncertain as errors. The labelled datasets were Metaxa2’s test file (50 SSU and 50 LSU sequences), 500 sequences sampled from the Metaxa2 database with 10 random negatives, the first 5000 reads of a ZymoBIOMICS Nanopore mock community (SRR10391201; all bacterial), and a simulated benchmark independent of the Metaxa2 database. For the last, sources for all five origins were drawn from SILVA 138.2 (Quast *et al.* 2013), with mitochondria and chloroplasts from the entries its taxonomy labels as such, excluding Metaxa2 accessions and stratified by identity to the closest Metaxa2 sequence, measured with VSEARCH (Rognes *et al.* 2016). Full-length reads were simulated from both strands at mean identities of 92%, 98.5% and 99.9% with Badread’s error models (Wick 2019). Supplementary Note S1 gives the settings, and Supplementary Tables S1–S3 the full confusion matrices, the reads on which the statistics disagree and the per-band results.

The summed score was the only statistic above 95% accuracy on every labelled real dataset (Table 1). The mean, which is also the default of Metaxa2’s HMM step, failed on the noisy Nanopore reads (43.0%; Metaxa2’s HMM step 54.9%): it sent 1826 bacterial reads to chloroplast and 955 to mitochondria. On the reads where the statistics disagreed, bacterial profiles matched a median of 9 regions against 4 for the organellar origins, at a slightly lower mean score, so a few strong organellar regions outscored broad bacterial support (Table S2). The sum errs the other way: organellar reads that also match many bacterial profiles can be called bacteria (19 of 100 chloroplast sequences in the reference set), consistent with Metaxa2 providing more bacterial (15) than chloroplast (12) or main-set mitochondrial (10) profiles. On the simulated reads, the summed score kept bacteria, archaea and eukaryota above 96% at every read accuracy and novelty band, whereas the mean assigned 21 to 57% of bacterial reads elsewhere (Table S3). Organelles were the summed score’s weak point: it recovered 62 to 78% of mitochondrial and 88 to 93% of chloroplast reads, losing most of the rest to bacteria, and chloroplast recall fell to 78 to 86% for sources below 97% identity to the Metaxa2 database. The mean recovered more organellar reads at 98.5% and 99.9% identity (85 to 86% of mitochondrial, 99.5% of chloroplast). Weighted to a bacteria-dominated or a plant-root-like composition, the summed score was 93 to 98% accurate, against 47 to 87% for the mean and 96 to 99% for Metaxa2 with BLAST. Metaxa2 with BLAST was the most accurate method on every labelled dataset, at a much higher cost in run time.

**Table 1:** Accuracy of origin assignment (%).

Dataset	SSU reads	Metaxa2		SSUplex		
		HMM	BLAST	Sum (default)	Mean	Count
**Metaxa2 test file**	50	96.0	100.0	96.0	96.0	66.0
**Metaxa2 reference set**	500	94.8	99.8	95.6	96.8	79.2
**ZymoBIOMICS Nanopore**	5000	54.9	99.96	95.9	43.0	95.7
**Simulated, 92% identity**	17 982	80.3	93.0	92.8	76.4	88.9
**Simulated, 98.5% identity**	17 982	91.6	94.3	90.7	89.5	70.8
**Simulated, 99.9% identity**	17 982	93.8	94.6	89.7	92.0	67.9

Reads a method leaves undetected, unclassified or uncertain count as errors. Metaxa2 HMM: origin from its HMM step; Metaxa2 BLAST: final origin after BLAST classification. Every method rejected all negatives in the real sets (50 LSU sequences in the test file; 10 random sequences in the reference set; at least 99.9% of the 4000 LSU and shuffled sequences in each simulated set). Simulated reads come from sources outside the Metaxa2 database, at the mean read identity shown; their accuracy is the mean of the five per-origin recalls, so that each origin counts equally (1982 mitochondrial and 4000 other reads per set). Results by origin and novelty band are in Table S3.

On 5000 reads of a rice-root Nanopore 16S sample (SRR25243163), which has no per-read truth, SSUplex assigned 3008 reads (60.2%) to bacteria, 1761 (35.2%) to chloroplast and 231 (4.6%) to mitochondria, and agreed with Metaxa2’s BLAST-assisted calls on 96.8% of reads (Metaxa2: 42.4% organellar). About two in five reads in this nominally bacterial 16S sample were organellar. The mean ranking would have reported 77.0%, largely by sending bacterial reads to organelles.

Performance was measured on the first 5000 to 1 000 000 ZymoBIOMICS reads with three replicates, 12 threads, Metaxa2 in extraction-only mode (-x T) and peak memory summed over each tool’s whole process tree (Fig. 2). From 200 000 reads upward SSUplex was 3.2× faster (550 against 1773 s at 1 000 000 reads) and used about 45% less peak memory (7.7 against 14.3 GB). Metaxa2’s full pipeline took 539 and 2865 s on 5000 and 25 000 reads, 65 and 134 times longer than SSUplex. Peak memory grows linearly with input for both tools, because reads are held in memory.

**Figure 2:**
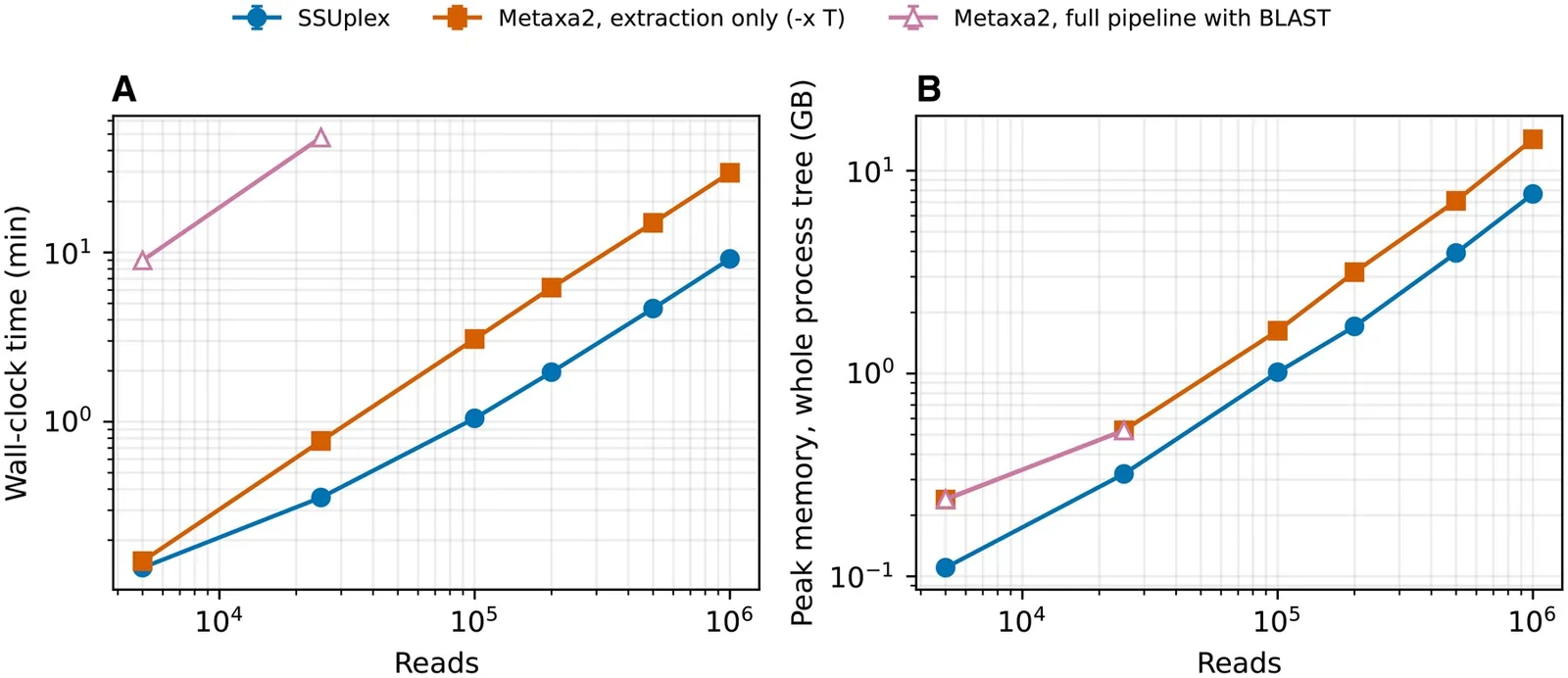
Run time (A) and peak memory (B) on the first 5000 to 1 000 000 reads of the ZymoBIOMICS Nanopore run (SRR10391201). Points are medians of three replicates, with the range as error bars (mostly smaller than the markers); 12 threads, AMD Ryzen 99,950X with 64 GB of memory, HMMER 3.4 for both tools. Metaxa2 ran extraction only (−x T); its full pipeline with BLAST classification was timed at 5000 and 25 000 reads. Peak memory is summed over each tool’s process tree; the full pipeline’s peak coincides with the extraction-only runs.

Two limitations follow from these results. First, organellar reads that also match many bacterial profiles can end up in the bacterial output (up to 38% of simulated mitochondrial and 12% of chloroplast reads were not recovered). Metaxa2 resolves many such reads with BLAST, whereas SSUplex leaves them to the downstream classifier, which labels them as chloroplast or mitochondria when its reference, like SILVA, includes organelle sequences; where the organellar fraction itself matters, —rank mean recovers more organellar reads from accurate reads, at the cost of misassigning bacterial ones. Second, the profiles inherit the coverage of the Metaxa2 database, built from SILVA release 111 and Mitozoa release 10; in the simulations, accuracy held for novel bacteria, archaea and eukaryota but fell for novel chloroplasts, and for novel mitochondria under Metaxa2’s BLAST step. SSUplex was designed and benchmarked for full-length long-read amplicons. It runs on short or shotgun reads, but a short read spans only one or two conserved regions, and those uses have not been tested.

Within this scope, SSUplex sorts long reads by origin within about five percentage points of Metaxa2’s full pipeline, at a small fraction of its run time, as a single tested binary that hands origin-sorted reads to marker-specific classifiers.

## Supplementary Material

vbag318_Supplementary_Data

## Data Availability

SSUplex v0.2.0 is available at https://github.com/ayobi/ssuplex under the MIT license and archived at Zenodo (O’Brien *et al.* 2026; https://doi.org/10.5281/zenodo.23246210). The scripts in the repository’s dev/directory regenerate every result reported here from public data, as described in dev/BENCHMARK.md. No new sequencing data were generated. Public data: the ZymoBIOMICS Nanopore mock community (SRR10391201; BioProject PRJNA587452), the rice-root Nanopore 16S sample (SRR25243163; BioProject PRJNA992961), SILVA release 138.2, and Metaxa2 2.2.3.
