# Supplementary material for: SSUplex: fast, both-strand extraction and origin-sorting of small-subunit rRNA for environmental DNA metabarcoding
Source: Bioinform Adv. 2026 Oct 23;6(1):vbag318. doi: 10.1093/bioadv/vbag318 (PMC13734377; doi:10.1093/bioadv/vbag318)
Supplement: Supplementary file 1 [file vbag318_supplementary_data.pdf]

Supplementary Material

SSUplex: fast, both-strand extraction and origin-sorting of small-subunit rRNA for environmental DNA metabarcoding

Aaron O'Brien, Juan Vargas Botia, Isabel Acuña, Franko Restovic, Patricio Martínez, Pilar Parada

Supplementary Note S1: Benchmark settings

All results were produced with the scripts in the `dev/` directory of the SSUplex repository at v0.2.0 (doi:10.5281/zenodo.23246210), as described in `dev/BENCHMARK.md`, on an AMD Ryzen 9 9950X with 64 GB of memory running Linux. SSUplex and Metaxa2 2.2.3 used the same HMMER 3.4 build; Metaxa2's BLAST classification used legacy BLAST 2.2.26. SSUplex used its HMM profiles assembled from the Metaxa2 2.2.3 SSU database (`dev/assemble_metaxa2_hmms.sh`). Accuracy runs used 16 threads, Metaxa2's default pipeline (which yields both its HMM-stage origin and its final origin) and SSUplex with `--rank sum`, `mean` and `count`.

Definitions. Accuracy is the number of correct calls divided by the number of reads with a known SSU origin; reads a method leaves undetected, unclassified or uncertain count as errors. Negatives (LSU sequences in the Metaxa2 test file, random sequences in the reference set) are scored separately as the fraction rejected. For the rice-root sample, which has no per-read truth, only agreement with Metaxa2's final calls is reported, among reads both methods assign to an SSU origin.

Datasets. Metaxa2 test file: `test.fasta` from the Metaxa2 2.2.3 distribution, 50 SSU sequences (ten per origin) and 50 LSU sequences, labelled from their identifiers. Reference set: 100 sequences per origin sampled from the Metaxa2 SSU database with seed 42, plus 10 random negatives (`dev/make_truth_set.py`). ZymoBIOMICS and rice-root reads: the first 5,000 reads of SRR10391201 and SRR25243163 (`dev/fetch_ena_reads.sh`).

Supplementary Table S1: Confusion matrices

Calls of each method on each labelled dataset, followed by the rice-root comparison. Metaxa2 HMM: origin from Metaxa2's HMM-based extraction step; Metaxa2 BLAST: Metaxa2's final origin after BLAST classification; SSUplex sum (the default), mean and count: SSUplex with each ranking statistic.

Metaxa2 test file. Rows: true origin; columns: call. Unc., uncertain (Metaxa2 only); Uncl., undetected or unclassified.

Method	True origin	Bact.	Arch.	Euk.	Mito.	Chlo.	Unc.	Uncl.
Metaxa2 HMM	Bact.	8			2			
	Arch.		10					
	Euk.			10				
	Mito.				10			
	Chlo.					10		
	Negative							50
Metaxa2 BLAST	Bact.	10						
	Arch.		10					
	Euk.			10				
	Mito.				10			
	Chlo.					10		
	Negative							50
SSUplex sum	Bact.	10						
	Arch.		10					
	Euk.			10				
	Mito.	1			9			
	Chlo.	1				9		
	Negative							50
SSUplex mean	Bact.	8			2			
	Arch.		10					
	Euk.			10				
	Mito.				10			
	Chlo.					10		
	Negative							50
SSUplex count	Bact.	10						
	Arch.		10					
	Euk.			10				
	Mito.	7			3			
	Chlo.	10						
	Negative							50

Metaxa2 reference set. Rows: true origin; columns: call. Unc., uncertain (Metaxa2 only); Uncl., undetected or unclassified.

Method	True origin	Bact.	Arch.	Euk.	Mito.	Chlo.	Unc.	Uncl.
Metaxa2 HMM	Bact.	80			19	1		
	Arch.		100					
	Euk.		1	97	2			
	Mito.	1			98			1
	Chlo.				1	99		
	Negative							10
Metaxa2 BLAST	Bact.	100						
	Arch.		100					
	Euk.			100				
	Mito.				99			1
	Chlo.					100		
	Negative							10
SSUplex sum	Bact.	100						
	Arch.		100					
	Euk.			100				
	Mito.				98			2
	Chlo.	19			1	80		
	Negative							10
SSUplex mean	Bact.	89			9	2		
	Arch.		99	1				
	Euk.			100				
	Mito.	1			97			2
	Chlo.				1	99		
	Negative							10
SSUplex count	Bact.	100						
	Arch.		100					
	Euk.	1		99				
	Mito.	3			95			2
	Chlo.	96			2	2		
	Negative							10

ZymoBIOMICS Nanopore, first 5,000 reads. Rows: true origin; columns: call. Unc., uncertain (Metaxa2 only); Uncl., undetected or unclassified.

Method	True origin	Bact.	Arch.	Euk.	Mito.	Chlo.	Unc.	Uncl.
Metaxa2 HMM	Bact.	2,745	20	286	1,070	877		2
Metaxa2 BLAST	Bact.	4,998						2
SSUplex sum	Bact.	4,795			69	124		12
SSUplex mean	Bact.	2,151	47	9	955	1,826		12
SSUplex count	Bact.	4,787			75	126		12

Rice-root Nanopore 16S sample (SRR25243163), first 5,000 reads. No per-read truth: reads assigned to each origin, the organellar share, and agreement with Metaxa2's BLAST-assisted calls among reads both methods assign to an SSU origin.

Method	Bact.	Arch.	Euk.	Mito.	Chlo.	Organellar (%)	Agreement (%)
Metaxa2 HMM	1,237	1	34	1,741	1,987	74.6	66.9
Metaxa2 BLAST	2,873	0	0	352	1,769	42.4	
SSUplex sum	3,008	0	0	231	1,761	39.8	96.8
SSUplex mean	1,141	7	2	1,602	2,248	77.0	64.5
SSUplex count	3,765	0	0	137	1,098	24.7	81.6

Supplementary Table S2: Reads on which the ranking statistics disagree

For every read on which `--rank mean`, `sum` and `count` assign different origins, grouped by the combination of calls. Truth gives the two most frequent true origins in each group (for the rice-root sample, Metaxa2's final call instead). For the origin chosen by the mean and the origin chosen by the sum, the table gives the median number of matched conserved regions, the median mean bit score and the median summed bit score over the reads in the group. Patterns seen in fewer than two reads are pooled as "other patterns".

Origin chosen by					Origin chosen by mean			Origin chosen by sum		
mean	sum	count	Reads	Truth (or Metaxa2 BLAST)	Regions	Mean	Sum	Regions	Mean	Sum
**Metaxa2 test file**: 19 of 100 reads										
Chlo.	Chlo.	Bact.	9	Chlo. 9	12	69.5	812	12	69.5	812
Mito.	Mito.	Bact.	6	Mito. 6	11	55.4	618	11	55.4	618
Mito.	Bact.	Bact.	3	Bact. 2, Mito. 1	7	57.6	404	12	50.8	627
other patterns			1							
**Metaxa2 reference set**: 114 of 510 reads										
Chlo.	Chlo.	Bact.	77	Chlo. 77	12	68.0	807	12	68.0	807
Chlo.	Bact.	Bact.	21	Chlo. 19, Bact. 2	11	56.5	623	14	49.1	664
Mito.	Bact.	Bact.	9	Bact. 9	9	55.7	520	14	54.4	742
Mito.	Mito.	Bact.	3	Mito. 3	11	55.4	610	11	55.4	610
other patterns			4							
**ZymoBIOMICS Nanopore, first 5,000 reads**: 2,806 of 5,000 reads										
Chlo.	Bact.	Bact.	1,715	Bact. 1,715	4	44.5	170	9	40.6	352
Mito.	Bact.	Bact.	930	Bact. 930	4	43.6	179	9	40.2	354
Arch.	Bact.	Bact.	46	Bact. 46	1	43.0	43	8	40.5	336
Bact.	Chlo.	Chlo.	29	Bact. 29	3	40.4	107	4	36.1	139
Bact.	Mito.	Mito.	28	Bact. 28	3	43.1	118	4	36.3	133
Chlo.	Mito.	Mito.	24	Bact. 24	2	43.3	87	4	36.1	144
Mito.	Chlo.	Chlo.	10	Bact. 10	2	43.0	86	4	37.1	149
Euk.	Bact.	Bact.	9	Bact. 9	1	44.1	44	10	41.2	420
Bact.	Bact.	Chlo.	5	Bact. 5	5	44.3	205	5	44.3	205
Bact.	Bact.	Mito.	3	Bact. 3	4	44.6	178	4	44.6	178
Chlo.	Bact.	Mito.	2	Bact. 2	3	47.1	141	6	45.9	274
other patterns			5							
**Rice-root sample (Metaxa2 BLAST call shown)**: 2,669 of 5,000 reads										
Mito.	Bact.	Bact.	1,389	Bact. 1,265, Mito. 124	8	51.5	408	13	47.7	610
Chlo.	Chlo.	Bact.	667	Chlo. 666, Bact. 1	12	60.6	721	12	60.6	721
Chlo.	Bact.	Bact.	481	Bact. 460, Chlo. 11	6	49.2	307	12	45.6	530
Mito.	Mito.	Bact.	93	Mito. 92, Bact. 1	8	51.9	415	8	51.9	415
Chlo.	Mito.	Mito.	12	Mito. 10, Bact. 2	3	47.4	138	8	44.2	313
Bact.	Mito.	Mito.	7	Mito. 5, Bact. 2	4	44.4	178	6	40.0	226
Arch.	Bact.	Bact.	6	Bact. 6	1	45.4	45	10	41.8	420
Bact.	Chlo.	Chlo.	5	Chlo. 3, Bact. 2	8	47.6	369	10	44.8	448
Bact.	Bact.	Chlo.	3	Bact. 2, Chlo. 1	8	50.6	405	8	50.6	405
Euk.	Bact.	Bact.	2	Bact. 2	1	46.8	47	14	43.6	588
other patterns			4							

Supplementary Table S3: Simulated benchmark

Reads simulated from sources outside the Metaxa2 database (`dev/sim/`). Sources: 2,000 per origin from SILVA 138.2 SSU Ref NR99 (bacteria, archaea and nuclear eukaryota by domain; mitochondria and chloroplasts from the entries SILVA's taxonomy labels as such), except mitochondria, for which all 991 unique sequences left after excluding Metaxa2 accessions were used (mainly plant, fungal and protist mitochondria). Sources were drawn evenly from three novelty bands where possible. Negatives: 1,000 LSU sequences from the Metaxa2 LSU database and 1,000 composition-preserving shuffles of SSU sources. Each source and negative gave two full-length reads from a random strand, with Badread's nanopore2020 (mean identity 92%), nanopore2023 (98.5%) and pacbio2021 (about 99.9%) error models: 21,982 reads per identity level. All methods rejected at least 99.9% of negatives.

(a) Recall (%) by read identity, true origin and novelty band. Band: identity of the source to its closest Metaxa2 database sequence; "all" pools the bands. Each source gave two reads.

				Metaxa2		SSUplex		
Read identity	Origin	Band (%)	Reads	HMM	BLAST	sum	mean	count
92%	bacteria	99–100	1,336	60.7	100.0	98.0	48.5	97.8
		97–99	1,332	51.4	100.0	96.9	40.5	97.0
		<97	1,332	48.5	98.5	96.3	40.1	96.2
		all	4,000	53.5	99.5	97.1	43.0	97.0
	archaea	99–100	1,336	85.0	99.9	99.6	83.9	99.6
		97–99	1,332	82.6	100.0	99.8	83.4	99.8
		<97	1,332	72.2	98.4	98.6	75.8	98.6
		all	4,000	80.0	99.4	99.3	81.0	99.3
	eukaryota	99–100	1,336	94.5	99.4	98.1	96.7	98.1
		97–99	1,332	94.1	99.8	98.1	96.8	98.1
		<97	1,332	92.6	99.5	96.8	95.8	96.8
		all	4,000	93.8	99.6	97.7	96.5	97.7
	mitochondria	99–100	238	79.0	80.3	81.1	75.6	73.5
		97–99	408	75.7	86.0	79.4	73.0	72.1
		<97	1,336	77.6	63.1	76.3	67.9	71.1
		all	1,982	77.4	69.9	77.5	69.9	71.6
	chloroplast	99–100	740	98.4	99.5	97.2	95.4	80.9
		97–99	1,928	98.2	99.2	95.4	93.1	81.4
		<97	1,332	94.4	91.1	85.8	87.6	74.0
		all	4,000	97.0	96.6	92.5	91.7	78.9
98.5%	bacteria	99–100	1,336	83.8	100.0	99.7	81.4	99.9
		97–99	1,332	73.6	100.0	99.5	66.9	99.9
		<97	1,332	69.9	99.0	99.4	61.4	99.9
		all	4,000	75.8	99.7	99.5	69.9	99.9
	archaea	99–100	1,336	98.1	100.0	100.0	97.2	100.0
		97–99	1,332	98.2	100.0	100.0	97.8	100.0
		<97	1,332	90.3	99.7	100.0	88.8	100.0
		all	4,000	95.5	99.9	100.0	94.6	100.0
	eukaryota	99–100	1,336	97.7	99.4	99.0	98.2	99.0
		97–99	1,332	97.0	99.8	99.8	99.3	99.8
		<97	1,332	96.5	99.7	98.7	98.0	98.7
		all	4,000	97.0	99.7	99.2	98.5	99.2
	mitochondria	99–100	238	85.3	85.3	65.1	81.9	38.7
		97–99	408	88.0	88.7	64.0	83.8	26.2
		<97	1,336	91.6	67.1	66.2	85.7	43.9
		all	1,982	90.1	73.7	65.6	84.9	39.6
	chloroplast	99–100	740	100.0	100.0	94.6	100.0	11.8
		97–99	1,928	99.7	99.9	93.4	99.8	11.6
		<97	1,332	98.9	95.7	79.4	98.6	22.5
		all	4,000	99.5	98.5	89.0	99.5	15.2
99.9%	bacteria	99–100	1,336	89.8	100.0	99.4	88.8	100.0
		97–99	1,332	79.6	100.0	99.7	77.3	100.0
		<97	1,332	75.4	99.1	99.4	70.0	100.0
		all	4,000	81.6	99.7	99.5	78.7	100.0
	archaea	99–100	1,336	99.4	100.0	100.0	99.0	100.0
		97–99	1,332	99.8	100.0	100.0	99.0	100.0
		<97	1,332	95.2	99.7	100.0	91.4	100.0
		all	4,000	98.2	99.9	100.0	96.5	100.0
	eukaryota	99–100	1,336	98.2	99.4	99.3	98.6	99.3
		97–99	1,332	98.0	99.8	99.8	99.4	99.8
		<97	1,332	95.7	99.7	99.0	98.6	99.0
		all	4,000	97.3	99.7	99.4	98.8	99.4
	mitochondria	99–100	238	88.2	85.7	58.4	80.7	36.6
		97–99	408	91.2	89.2	56.4	86.0	19.9
		<97	1,336	93.4	68.5	63.8	87.4	37.9
		all	1,982	92.3	74.8	61.6	86.3	34.1
	chloroplast	99–100	740	100.0	100.0	93.4	100.0	4.1
		97–99	1,928	99.7	100.0	93.1	99.8	2.0
		<97	1,332	98.9	96.8	78.2	98.8	13.3
		all	4,000	99.5	98.9	88.2	99.5	6.1

(b) Expected accuracy (%) and reported organellar share (%) for two sample compositions. Computed from the per-origin call rates in (a), which do not depend on composition. Bacteria-dominated: 90% bacteria, 2% archaea, 2% eukaryota, 3% mitochondria, 3% chloroplast (true organellar share 6%). Plant-root-like: 55% bacteria, 1% archaea, 2% eukaryota, 7% mitochondria, 35% chloroplast (42%), close to Metaxa2's BLAST-assisted calls on the rice-root sample.

		Metaxa2		SSUplex		
Read identity	Composition, measure	HMM	BLAST	sum	mean	count
92%	bacteria-dominated, accuracy	56.9	98.5	96.4	47.1	95.8
	bacteria-dominated, organellar (true 6)	42.8	5.2	7.9	55.4	7.4
	plant-root-like, accuracy	71.5	96.4	94.2	63.4	88.9
	plant-root-like, organellar (true 42)	63.3	39.2	39.9	69.9	34.7
98.5%	bacteria-dominated, accuracy	77.8	98.9	98.2	72.3	95.6
	bacteria-dominated, organellar (true 6)	26.1	5.4	5.2	32.1	1.7
	plant-root-like, accuracy	85.7	97.5	93.5	82.1	66.1
	plant-root-like, organellar (true 42)	54.0	40.1	36.3	57.6	8.3
99.9%	bacteria-dominated, accuracy	83.1	98.9	98.0	80.3	95.2
	bacteria-dominated, organellar (true 6)	21.3	5.4	5.1	24.2	1.2
	plant-root-like, accuracy	89.1	97.7	92.9	87.1	62.5
	plant-root-like, organellar (true 42)	51.2	40.4	35.8	52.8	4.6

Supplementary Note S2: Use of AI tools

This note documents the use of AI tools, as required by the journal's policy.

Original development (2026). Claude (Anthropic) and ChatGPT (OpenAI) were used for code review, writing and editing assistance, and GitHub Copilot (Microsoft) for code completion.

Revision. Claude (Anthropic), used through claude.ai, was used to:

- write the benchmark, timing and simulation scripts in `dev/`, and the script that produced these supplementary tables;
- propose and implement code changes in SSUplex: overwrite protection (`--force`), passing `--dna` to `nhmmer`, the change of the default ranking statistic to the summed score, corrected help texts, and the declared minimum Rust version;
- rewrite the README and write `dev/BENCHMARK.md`;
- redraw Figure 1 and write the plotting code for Figure 2;
- draft revised manuscript text, which the corresponding author then rewrote in his own words, and draft the response to reviewers, which he revised.

The authors decided on every change, including the new default, ran all benchmarks on their own hardware, and reviewed all code, documentation and text; the corresponding author rewrote the manuscript text in his own words. The history of each code change, with its description, is in the repository's version control history.
